# Prognostic value of carbonic anhydrase VII expression in colorectal carcinoma

**DOI:** 10.1186/s12885-015-1216-y

**Published:** 2015-04-01

**Authors:** Guang-Zhen Yang, Liang Hu, Jian Cai, Hai-Yang Chen, Yu Zhang, Dan Feng, Chen-Ye Qi, Yan-Xia Zhai, Hui Gong, Hao Fu, Qing-Ping Cai, Chun-Fang Gao

**Affiliations:** 1 Anal-Colorectal Surgery Institute, 150th Hospital of PLA, Luoyang, China; 2 Department of Clinical Laboratory, 150th Hospital of PLA, Luoyang, China; 3 Department of Colorectal Surgery, 150th Hospital of PLA, Luoyang, China; 4 Department of Oncology, 150th Hospital of PLA, Luoyang, China; 5 Department of Oncology, Changhai Hospital, Second Military Medical University, Shanghai, China; 6 Department of Gastrointestine Surgery, Changzheng Hospital, Second Military Medical University, Shanghai, China

**Keywords:** CA7, Colorectal carcinoma, Survival, Prognosis, Early stage

## Abstract

**Background:**

Carbonic anhydrases (CAs) have been implicated in the pathogenesis of human cancers. Carbonic anhydrase VII (CA7), a member of the CA gene family, was recently demonstrated to be expressed in several human tissues including colon. Nevertheless, the expression and clinical relevance of CA7 in colorectal carcinoma (CRC) has not been investigated.

**Methods:**

Real-time PCR, western blot, and immunohistochemistry analyses were used to determine CA7 expression in CRC clinical samples. The correlation of CA7 expression with clinicopathologic features was assessed in 228 patients from Luoyang, China (training cohort) and validated in 151 patients from Shanghai, China (validation cohort). Kaplan-Meier and Cox proportional regression analyses were used to estimate the association between CA7 expression and patients’ survival.

**Results:**

CA7 expression was frequently downregulated in CRC tissues at both the mRNA and protein levels. Reduced expression of CA7 was significantly correlated with poor differentiation, positive lymph node metastasis, advanced TNM stage and unfavorable clinical outcome not only in the training cohort but also in the validation set. Survival analysis indicated that patients with lower CA7 expression had a significantly shorter disease-specific survival (DSS) than those with higher CA7 expression. Importantly, further stage-based analyses revealed that decreased CA7 expression significantly predicted poor DSS and was an independent adverse prognostic indicator for patients with early stage tumors in both cohorts.

**Conclusions:**

Our results indicate that decreased expression of CA7 correlates with disease progression and predicts poor prognosis in CRC, especially for patients with early stage tumors.

**Electronic supplementary material:**

The online version of this article (doi:10.1186/s12885-015-1216-y) contains supplementary material, which is available to authorized users.

## Background

Colorectal carcinoma (CRC) is one of the leading causes of cancer-related death globally, accounting for more than 1.2 million new cases and 600,000 deaths per year [1,2]. Although the survival of patients with CRC has slowly but steadily improved during the past decades in the developed countries, mortality rates have continued increasing in countries including China [3,4]. Due to post-surgical recurrence and fatal distant metastasis, the prognosis for CRC patients has shown only limited improvement despite advances in treatment approaches over the last few years. Therefore, it is urgent needed to search for valuable biomarkers to improve prognosis prediction and clinical outcome of patients with CRC.

Carbonic anhydrases (CAs) are a family of ubiquitously expressed metalloenzymes that catalyze the reversible conversion of carbon dioxide to bicarbonate and proton [5]. Previously studies have revealed that CAs are involved in multiple physiological and pathological processes including gluconeogenesis, lipogenesis, ureagenesis and tumorigenicity [6]. In humans, at least 15 CA isozymes with different catalytic activity, subcellular localization and tissue distribution have been described [5]. Among them, aberrant expression of CA I, II, IX, XII and XIII has been reported in CRC [7-12]. Recently, CA7, a cytosolic isoform of CAs with high carbon dioxide hydration activity, was demonstrated to be expressed in several normal tissues including colon [13]. A previous work from a gene expression microarray analysis revealed that CA7 was downregulated in clinically left sided colon tumors [14]. More recently, a bioinformatics-based study indicated CA7 as an important suppressor gene for the classification of normal and CRC tissues [15]. In addition, it has been shown that upregulated expression of CA7 was associated with poor prognosis of patients with astrocytomas [16].

Despite these, to our knowledge, systematic investigation of the expression and clinical implications of CA7 in human CRC has not been reported. In the present study, we examined the expression of CA7 in CRC clinical samples and assessed the correlation of CA7 expression with clinicopathologic features and with patient survival in a training cohort and further validated our findings in an independent external cohort. Our data demonstrated that decreased expression of CA7 could serve as an independent predictor of poor prognosis in CRC, especially for patients who have early stage tumors.

## Methods

### Patients and tissue samples

We obtained pathologically confirmed formalin-fixed paraffin-embedded tissue specimens of 379 stages I–III CRC patients with typical adenocarcinoma histology. Of these, 228 received curative surgery in 150th Hospital of PLA (Luoyang, China) between May 2006 and October 2008 and 151 received curative surgery in Changzheng Hospital, Second Military Medical University (Shanghai, China) between July 2006 and April 2008. Distribution of the continuous variables of the two study cohorts was listed in Additional file 1. Detailed clinicopathologic features of CRC patients were listed in Table 1. The follow-up period was defined as the interval from the date of surgery to the date of death or last follow-up. The final date of follow-up was 26 September 2014 for patients from 150th Hospital of PLA (the Luoyang cohort) and 11 July 2014 for patients from Changzheng Hospital (the Shanghai cohort). Disease-specific survival (DSS) was defined as the interval from the date of surgery to the date that patient died of CRC. Patients alive at the end of follow-up were treated as censored data. Patients were excluded from the study cohorts with the following exclusion criteria: previously received any anticancer therapy; impaired heart, lung, liver, or kidney function; previous malignant disease. TNM staging was classified according to the American Joint Committee on Cancer staging manual (seventh edition).

**Table 1:** **Clinicopathologic features of CRC patients in the training and validation cohorts**

Characteristics	Training cohort (n = 228)		Validation cohort (n = 151)	
	No. of patients (%)		No. of patients (%)	
**Age (years)**				
<60	71	(31.1)	43	(28.5)
≥60	157	(68.9)	108	(71.5)
**Sex**				
Female	95	(41.7)	61	(40.4)
Male	133	(58.3)	90	(59.6)
**Tumor location**				
Rectum	169	(74.1)	81	(53.6)
Colon	59	(25.9)	70	(46.4)
**Differentiation grade**				
Well/Moderate	171	(75.0)	114	(75.5)
Poor	57	(25.0)	37	(24.5)
**Tumor size (cm)**				
<5	92	(40.4)	57	(37.7)
≥5	136	(59.6)	94	(62.3)
**Local invasion**				
T_1_-T_2_	44	(19.3)	14	(9.3)
T_3_-T_4_	184	(80.7)	137	(90.7)
**Lymph node metastasis**				
N_0_	132	(57.9)	84	(55.6)
N_1_	64	(28.1)	48	(31.8)
N_2_	32	(14.0)	19	(12.6)
**TNM stage**				
I	36	(15.8)	10	(6.6)
II	96	(42.1)	74	(49.0)
III	96	(42.1)	67	(44.4)
**Death**				
No	124	(54.4)	82	(54.3)
Yes	104	(45.6)	69	(45.7)

Fresh-frozen CRC samples obtained from 84 stages I–III primary CRC patients who received curative surgery in 150th Hospital of PLA from April 2013 to September 2013 were used for quantitative polymerase chain reaction (qPCR) and Western blot analysis. Written informed consent was obtained from each patient and this study was approved by the Ethical Committee of 150th Hospital of PLA and Changzheng Hospital.

### Real-Time qPCR analysis

Real-Time qPCR analysis was performed as described previously [17]. Briefly, total RNAs were isolated from frozen specimens using TRIzol Reagent (Invitrogen). Reverse transcription was performed using RevertAid™ First Strand cDNA Synthesis Kit (Thermo Scientific) according to the manufacturer’s instructions. qPCR was performed on ABI Prism 7500 Sequence Detection System with SYBR Premix Ex Taq™ II (Takara) using the 2^-ΔΔCT^ method. Gene expression results were normalized by internal control β-actin. The primers used in this study are as follows: CA7 (NM_005182.2) forward, 5'-CTGCTTTAAGAGGCTGCTCCG-3'; reverse, 5'-CCCTGGGCAATGGGATACAG-3'; β-actin (NM_001101.3) forward, 5'-AATCGTGCGTGACATTAAGGAG-3'; reverse, 5’-ACTGTGTTGGCGT ACAGGTCTT-3'. Each sample was tested in triplicate.

### Western blot analysis

Western blotting was performed as described previously [18]. Briefly, tumor specimens were prepared in lysis buffer [Tris–HCl (20 mM), pH 7.4, NaCl (150 mM), glycerol (10%), Nonidet P-40 (0.2%), EDTA (1 mM), EGTA (1 mM), PMSF (1 mM), NaF (10 mM), aprotinin (5 mg/ml), leupeptin (20 mM), and sodium orthovanadate (1 mM)] and centrifuged at 12,000 g for 30 min. Protein concentrations were measured using the BCA assay. Immunoblotting was performed using a primary antibody specific for CA7 (Abcam, ab103116) and immunocomplexes were incubated with a goat anti-rabbit fluorescein- conjugated secondary antibody, and then detected using an Odyssey fluorescence scanner (Li-Cor, Gene Company). β-actin was used as a loading control (Santa Cruz Biotechnology, sc-47778).

### Immunohistochemistry analysis

Immunohistochemistry of paraffin-embedded tissue sections was performed as described previously [19]. Briefly, sections were deparaffinized and rehydrated. The endogenous peroxidase activity was blocked with 3% H_2_O_2_ for 10 minutes. Antigens were retrieved with citrate buffer (10 mM, pH 6.0) for 15 minutes at 100°C in a microwave oven. After blocking, the sections were incubated with a primary anti-CA7 antibody (Abcam, ab103116) with 1:50 dilution at 4°C overnight in a moist chamber followed by incubated with an anti-rabbit peroxidase-conjugated secondary antibody (Santa Cruz) at room temperature for 30 minutes. Finally, the visualization signal was developed with diaminobenzidine (Dako) and the slides were counterstained with hematoxylin.

Stained sections were evaluated in a blinded manner without prior knowledge of the clinical data using the German immunoreactive score (IRS) as described previously [20]. Briefly, staining intensity was graded as “0” (negative), “1” (weak), “2” (moderate) and “3” (strong); staining extent was graded as “0” (<5%), “1” (5-25%), “2” (25-50%), “3” (50-75%) or “4” (>75%). Values of the staining intensity and the staining extent were multiplied as a final IRS of CA7 expression. Intratumoral CA7 expression was defined as follows: low expression with the IRS < 3 and high expression with the IRS ≥ 3. Discrepancies in the IRS were resolved by discussing together with other pathologists to reach a consensus. Tissue samples of patients from the Luoyang cohort were used as a training set. Prognostic value of the expression of CA7 was subsequently validated in the patients from the Shanghai cohort as an external validation set.

### Statistical analysis

Mann–Whitney U test was used to compare CA7 levels between groups. Pearson chi-square test or Fisher exact test was used to analyze the relationship between CA7 expression and clinical features. Kaplan-Meier analysis with log-rank test was used to compare patients’ survival between subgroups. The effect of each variable on survival was determined by the Cox multivariate regression analysis. All statistical analyses were carried out using SPSS PASW Statistics 18.0 software (SPSS, Inc., Chicago, IL), and *p* value < 0.05 were considered to be statistically significant.

## Results

### 1. Downregulation of CA7 in CRC tissues

**Figure 1:**
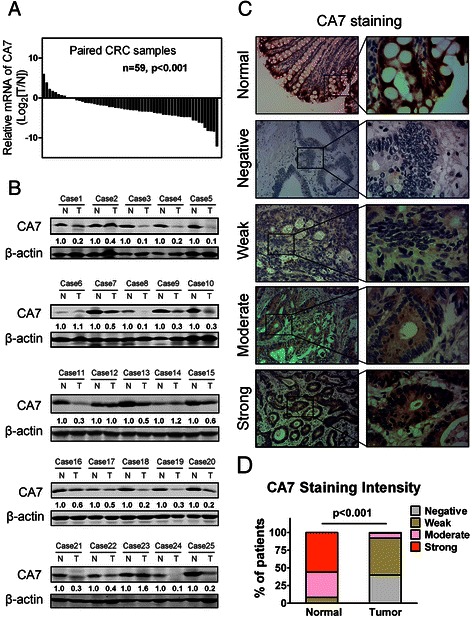
**CA7 expression is frequently downregulated in CRC. (A)** The expression levels of CA7 mRNA in 59 paired human primary CRC tissues (T) and adjacent normal tissues (N) were evaluated by real-time qPCR methods. (T vs N, p < 0.001) **(B)** Protein levels of CA7 in an independent set of 25 paired CRC specimens and adjacent normal tissues were determined by Western blot assay. β-actin was used as a loading control. The relative protein expression of CA7 was quantified and normalized to β-actin. Each N was arbitrarily designated 1.0. (T: Tumor; N: adjacent normal tissues, T vs N, p < 0.001) **(C)** Representative immunohistochemical expression patterns of CA7 in cancerous and adjacent normal mucosa specimens were shown. (Magnification, left panel, ×100; right panel, ×400) **(D)** Percentage of patients with different staining intensity of CA7 in the tumor or adjacent normal tissues in the training cohort (p < 0.001).

We first examined the expression levels of CA7 mRNA in 59 paired human primary CRC tissues and adjacent normal mucosa tissues using real-time qPCR analysis. As shown in Figure 1A, CA7 mRNA expression was markedly decreased in tumor tissues compared with adjacent normal mucosa tissues (50/59, p < 0.001). In addition, Western blot analysis from an independent set of 25 paired CRC and adjacent normal specimens demonstrated that protein expression of CA7 was significantly downregulated in tumor tissues compared with adjacent normal tissues (18/25, p < 0.001, Figure 1B).

To further investigate the phenotypic expression patterns of CA7 protein in CRC tissues, IHC analysis was performed in 228 specimens of patients from the training cohort. Representative patterns of CA7 expression (negative, weak, moderate, strong) were shown in Figure 1C. Positive staining of CA7 was mainly localized in the cytoplasm. Of note, in normal colon, the strongest immunostaining for CA7 was predominantly localized in the superficial part of the mucosa, while its expression in carcinomas was diffuse. Overall, 56.1% (128/228) of the adjacent normal mucosa tissues presented strong immunostaining, 35.6% (81/228) of cases showed moderate staining, 8.3% (19/228) showed weak staining and none showed negative staining of CA7 protein. In striking contrast, 39.9% (91/228) of the cancerous tissues investigated showed negative immunoreactivity, 52.2% (119/228) of cases showed weak staining, 7.0% (16/228) showed moderate staining and only 0.9% (2/228) showed relatively strong staining of CA7 (p < 0.001, Figure 1D). Consistently, IHC data from the validation cohort containing 151 CRC patients yielded a similar result (Additional file 2). Thus, the significant decreased staining signal for CA7 in the cancerous specimens definitely confirmed that CA7 was frequently downregulated in CRC tissues.

### 2. Correlation of CA7 expression with clinicopathologic features

Next, we evaluated the relationship between CA7 expression levels and clinicopathologic characteristics of CRC patients. Based on the immunoreactive score (IRS) of intratumoral CA7 expression, patients in the training cohort were divided into high and low CA7 expression subgroups with the median IRS value as the cut-off. As shown in Table 2, low expression of CA7 protein was significantly correlated with poor differentiation (p = 0.006), positive lymph node metastasis (p = 0.003), advanced TNM stage (p = 0.008) and increased death (p < 0.001). No significant associations were observed between CA7 expression and patient age, sex, tumor location, tumor size or local invasion.

**Table 2:** **Association between CA7 expression and clinicopathologic characteristics of CRC patients in the training and validation cohorts**

	Training cohort (n = 228)					Validation cohort (n = 151)				
Characteristics	CA7 expression				P value^a^	CA7 expression				P value^a^
	Low (%)		High (%)			Low (%)		High (%)		
	(n = 116)		(n = 112)			(n = 89)		(n = 62)		
**Age (years)**					0.267					0.622
<60	40	(34.5)	31	(27.7)		24	(27.0)	19	(30.6)	
≥60	76	(65.5)	81	(72.3)		65	(73.0)	43	(69.4)	
**Sex**					0.720					0.490
Female	47	(40.5)	48	(42.9)		38	(42.7)	23	(37.1)	
Male	69	(59.5)	64	(57.1)		51	(57.3)	39	(62.9)	
**Tumor location**					0.224					0.806
Rectum	90	(77.6)	79	(70.5)		47	(52.8)	34	(54.8)	
Colon	26	(22.4)	33	(29.5)		42	(47.2)	28	(45.2)	
**Differentiation grade**					**0.006**					**0.046**
Well/Moderate	78	(67.2)	93	(83.0)		62	(69.7)	52	(83.9)	
Poor	38	(32.8)	19	(17.0)		27	(30.3)	10	(16.1)	
**Tumor size (cm)**					0.448					0.586
<5	44	(37.9)	48	(42.9)		32	(36.0)	25	(40.3)	
≥5	72	(62.1)	64	(57.1)		57	(64.0)	37	(59.7)	
**Local invasion**					0.141					0.064
T_1_-T_2_	18	(15.5)	26	(23.2)		5	(5.6)	9	(14.5)	
T_3_-T_4_	98	(84.5)	86	(76.8)		84	(94.4)	53	(85.5)	
**Lymph node metastasis**					**0.003**					**0.003**
N_0_	56	(48.3)	76	(67.9)		41	(46.1)	43	(69.4)	
N_1_	36	(31.0)	28	(25.0)		31	(34.8)	17	(27.4)	
N_2_	24	(20.7)	8	(7.1)		17	(19.1)	2	(3.2)	
**TNM stage**					**0.008**					**0.003**
I	13	(11.2)	23	(20.5)		2	(2.2)	8	(12.9)	
II	43	(37.1)	53	(47.3)		39	(43.9)	35	(56.5)	
III	60	(51.7)	36	(32.2)		48	(53.9)	19	(30.6)	
**Death**					**<0.001**					**<0.001**
No	47	(40.5)	77	(68.8)		37	(41.6)	45	(72.6)	
Yes	69	(59.5)	35	(31.2)		52	(58.4)	17	(27.4)	

^a^Pearson chi-square test or Fisher exact test was used for comparison between subgroups. Bold type indicates statistical significance.

We then applied the same cut-off to dichotomise the study patients in the validation cohort. Consistently, low levels of CA7 protein were significantly correlated with differentiation grade (p = 0.046), lymph node metastasis (p = 0.003), TNM stage (p = 0.003) and patient death (p < 0.001). Collectively, these results indicated that intratumoral CA7 expression was negatively associated with the progression of CRC.

### 3. Prognostic values of CA7 expression in CRC patients

**Figure 2:**
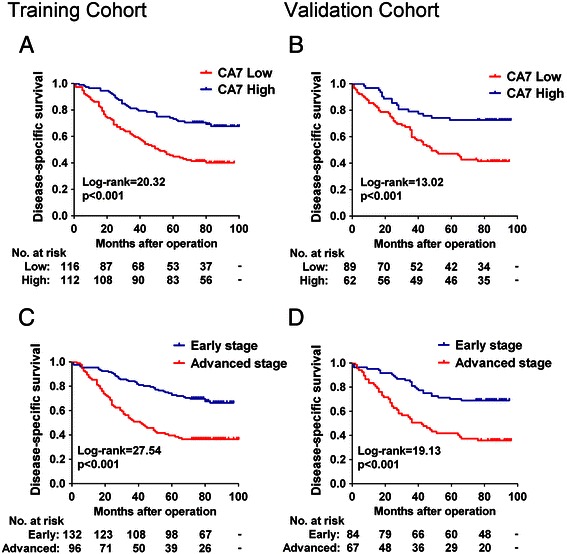
**Kaplan-Meier survival analysis for CRC patients in the training and validation cohorts. (A-B)** Kaplan-Meier curves for disease-specific survival of CRC patients in the training **(A)** and validation **(B)** cohorts according to CA7 expression status. Patients were divided into high and low CA7 expression subgroups with the median IRS value as the cut-off. **(C-D)** Kaplan-Meier curves for disease-specific survival of CRC patients in the training **(C)** and validation **(D)** cohort according to TNM stage of the disease. The p-value was determined using the log-rank test.

**Figure 3:**
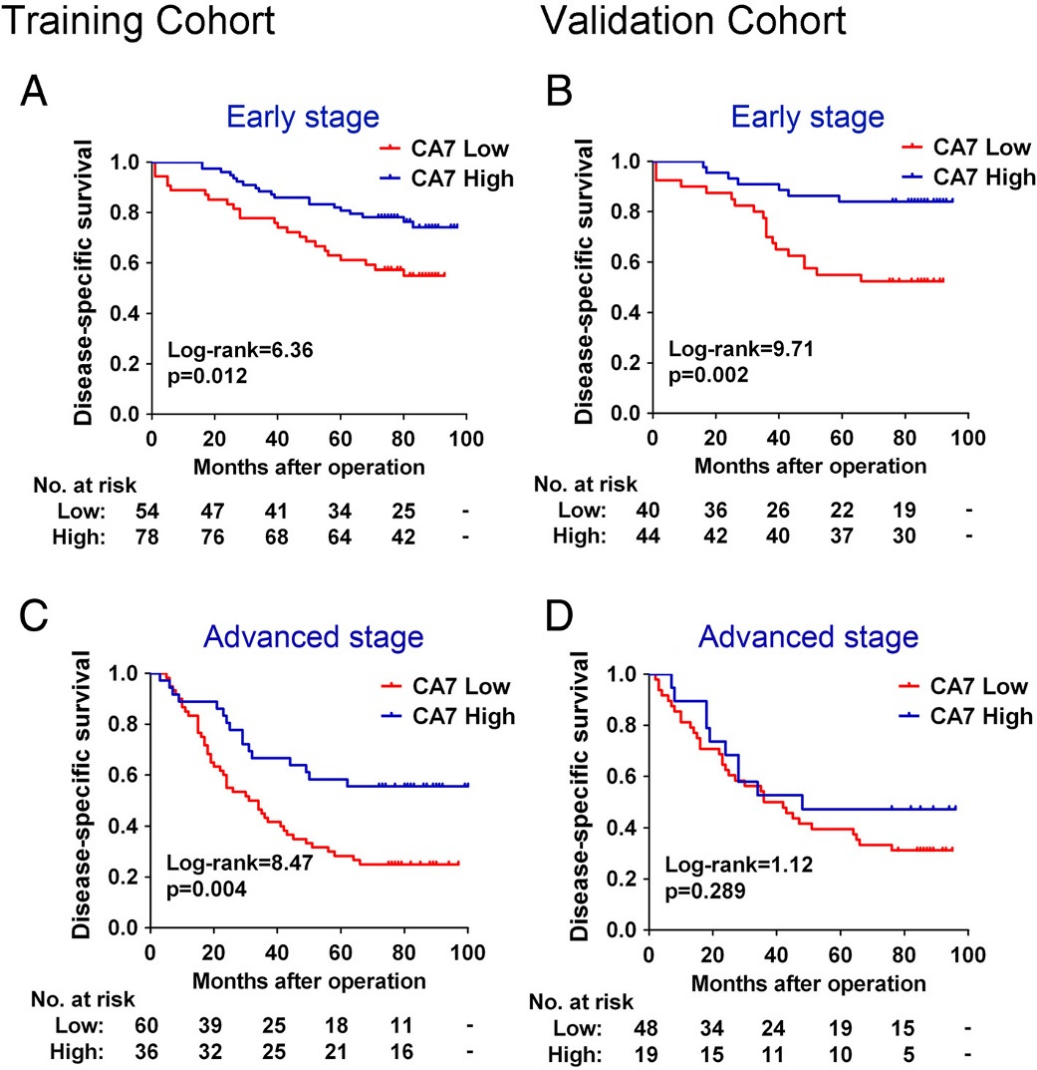
**Kaplan-Meier survival analysis for CRC patients with early or advanced stage tumors in the training and validation cohorts. (A-B)** Kaplan-Meier curves for disease-specific survival of CRC patients with early stage tumors in the training **(A)** and validation **(B)** cohorts according to CA7 expression status. **(C-D)** Kaplan-Meier curves for disease-specific survival of CRC patients with advanced stage tumors in the training **(C)** and validation **(D)** cohorts according to CA7 expression status. The p-value was determined using the log-rank test.

Kaplan-Meier survival analyses showed that patients with low CA7 expression had significantly poorer DSS rates than those with high CA7 expression in the training cohort (p < 0.001, Figure 2A). The cumulative 5-year DSS rate was 73.2% for patients in the high-CA7-expression group, whereas it was only 44.8% for those in the low-CA7-expression group. Similarly, patients who had tumors with low CA7 expression had a significantly shorter DSS than those who had tumors with high CA7 expression in the validation cohort (p < 0.001, Figure 2B). In both of the two cohorts, patients who had advanced stage (stage III) tumors had a significantly worse prognosis than those who had early stage (stages I-II) tumors (all p < 0.001, Figure 2C and D). These data were consistent with the well established adverse prognostic effect of tumor stage [21] and confirmed that our cohorts were representative and that the survival analyses were valid. Importantly, reduced expression of CA7 significantly predicted poor DSS for patients with early stage tumors both in the training (p = 0.012, Figure 3A) and validation cohorts (p = 0.002, Figure 3B). In addition, low levels of CA7 protein also predicted unfavorable DSS for patients with advanced stage tumors in the training cohort (p = 0.004, Figure 3C). While, the survival difference was not statistically significant for patients with advanced stage tumors in the validation cohort (p = 0.289, Figure 3D).

The independent prognostic significance of CA7 expression on CRC-specific survival was assessed with a Cox regression model. The clinicopathologic variables considered to be potential predictors of survival were shown in Table 3. Univariate analyses indicated that factors including patient age, tumor differentiation grade, TNM stage and CA7 expression were predictors of DSS both in the training and validation cohort. The factors that significantly correlated with survival in the univariate analysis were further assessed by multivariate analysis. The results revealed that, besides the patient age and TNM stage of the disease, decreased CA7 expression was an independent adverse prognostic factor not only in the training cohort (HR, 2.247; 95% CI, 1.481-3.401, p < 0.001) but also in the validation cohort (HR, 2.058; 95% CI, 1.174-3.610, p = 0.012).

**Table 3:** **Univariate and multivariate analyses of CA7 expression and disease-specific survival of patients in the training and validation cohorts**

Variables	Categories	Univariate analysis			Multivariate analysis^b^		
		HR	95% CI	P value^c^	HR	95% CI	P value^c^
**Training Cohort**							
Age (years)	≥60 / <60	2.263	1.389-3.687	**0.001**	2.770	1.691-4.537	**<0.001**
Sex	Male / female	0.878	0.596-1.293	0.510			
Tumor location	Colon / rectum	1.401	0.923-2.125	0.113			
Tumor size (cm)	≥5 / <5	1.114	0.753-1.648	0.590			
Differentiation grade	Poor / well + moderate	2.626	1.761-3.916	**<0.001**	2.010	1.337-3.023	**0.001**
TNM stage	III / I+ II	2.734	1.846-4.048	**<0.001**	2.402	1.603-3.598	**<0.001**
CA7 expression^a^	Low / high	2.469	1.642-3.717	**<0.001**	2.247	1.481-3.401	**<0.001**
**Validation Cohort**							
Age (years)	≥60 / <60	1.976	1.080-3.614	**0.027**	2.293	1.247-4.217	**0.008**
Sex	Male / female	0.737	0.459-1.183	0.207			
Tumor location	Colon / rectum	0.961	0.599-1.542	0.868			
Tumor size (cm)	≥5 / <5	0.814	0.503-1.317	0.403			
Differentiation grade	Poor / well + moderate	1.688	1.017-2.803	**0.043**			
TNM stage	III / I+ II	2.828	1.734-4.611	**<0.001**	2.674	1.615-4.428	**<0.001**
CA7 expression^a^	Low / high	2.632	1.520-4.545	**0.001**	2.058	1.174-3.610	**0.012**

*Abbreviations: HR* hazard ratio, *95% CI* 95% confidence interval.

^a^For CA7, median values were used as the cut-off point for definition of subgroups (low expression and high expression groups).

^b^Multivariate models were adjusted for age, sex, tumor location, tumor size, differentiation grade, and TNM stage.

^c^Bold type indicates statistical significance.

More importantly, stage-based survival analyses revealed that decreased expression of CA7 also was an independent predictor of poor prognosis for patients with early stage tumors in the training cohort (HR, 2.232; 95% CI, 1.220-4.082, p = 0.009) as well as in the validation cohort (HR, 3.165; 95% CI, 1.326-7.519, p = 0.009) (Table 4). In addition, low CA7 expression was also independently associated with poor DSS for patients with advanced stage tumors in the training cohort (HR, 2.809; 95% CI, 1.524-5.181, p = 0.001). However, the prognostic significance of CA7 was not statistically significant for patients with advanced stage tumors in the validation cohort. Taken together, these data demonstrated that decreased CA7 expression was closely related to poor patient survival, especially for those with early stage tumors.

**Table 4:** **Multivariate analyses of CA7 expression and disease-specific survival for patients with early or advanced stage tumors in the training and validation cohorts**

Variables	Categories	Early stage			Advanced stage		
		HR	95% CI	P value^b^	HR	95% CI	P value^b^
**Training Cohort**							
Age (years)	≥60 / <60	4.062	1.592-10.365	**0.003**	2.353	1.282-4.319	**0.006**
Sex	Male / female						
Tumor location	Colon / rectum						
Tumor size (cm)	≥5 / <5						
Differentiation grade	Poor / well + moderate				2.389	1.419-4.023	**0.001**
CA7 expression^a^	Low / high	2.232	1.220-4.082	**0.009**	2.809	1.524-5.181	**0.001**
**Validation Cohort**							
Age (years)	≥60 / <60				2.374	1.176-4.794	**0.016**
Sex	Male / female						
Tumor location	Colon / rectum						
Tumor size (cm)	≥5 / <5						
Differentiation grade	Poor / well + moderate				2.637	1.414-4.920	**0.002**
CA7 expression^a^	Low / high	3.165	1.326-7.519	**0.009**			

*Abbreviations:HR* hazard ratio, *95% CI* 95% confidence interval.

^a^For CA7, median values were used as the cut-off point for definition of subgroups (low expression and high expression groups).

^b^Bold type indicates statistical significance.

Multivariate models were adjusted for age, sex, tumor location, tumor size and differentiation grade.

## Discussion

To date, CAs have been implicated in the tumorigenesis of several human malignancies including CRC during the past decades. It has been shown that CA I, II and XIII are downregulated while CA IX and XII are upregulated in the cancerous tissue compared with the normal colorectal epithelium [7-12]. In addition, prognostic implications of individual CA isozymes in CRC have also been demonstrated. For instance, CA I positive immunostaining has been associated with well or moderate differentiation, lack vascular invasion and favorable clinical outcome of CRC [7,22], whereas, high expression of CA IX has been linked with worse prognosis of CRC patients [23-25]. Thus, distinct tumor-associated CAs apparently have different expression patterns and prognostic significances in CRC.

Recently, attention has been focus on the CA7 because of its high catalytic activity and relatively limited tissue distribution [13,26]. It has been shown that CA7 is expressed in several organs including brain, stomach, duodenum, colon, liver, and skeletal muscle. In addition, the expression of CA7 and its prognostic significance has been demonstrated in human diffuse astrocytomas [16]. However, its clinical relevance has not been assessed in CRC. Although a previous gene expression profiling study and a recent bioinformatics-based analysis revealed that CA7 mRNA was downregulated in CRC clinical specimens [14,15], neither of the two studies conducted validation experiments. To get a better insight into the phenotypic expression and prognostic significance of CA7 in CRC, we comprehensively analyzed both colorectal neoplasias and matched adjacent normal mucosa specimens from the same patients in two independent study cohorts.

Using qPCR and Western blot analysis, we demonstrated that CA7 was significantly downregulated in primary CRC samples at both the mRNA and protein levels. Strikingly, subsequent immunohistochemical analysis of CRC specimens from the training and validation cohorts showed that 56.1% and 66.9% of the adjacent normal mucosa sections presented strong CA7 immunoreactivity, whereas only 0.9% and 1.9% of the CRC sections showed relatively strong staining, respectively. These results definitely confirmed the significant downregulation of CA7 in CRC. Thus, the expression pattern of CA7 was generally similar with that of CA I, II and XIII in CRC [8,12]. While, the molecular basis for CA7 downregulation in CRC remains unclear and requires further investigation.

In the present study, we observed that the strongest CA7 immunoreaction was localized primarily in the mature superficial enterocytes but the signals reduced significantly along with increasing malignancy grades, indicating an association between CA7 expression and the differentiation of colorectal epithelium. In fact, several CA isozymes have been proposed to relate to differentiation. Bekku S *et al.* showed that CA I and II could be a differentiation marker of human and rat colonic enterocytes [27]. In addition, Leppilampi M *et al*. revealed that high expression of CA IX was associated with a differentiated phenotype of gastric epithelial cells [28]. Although our data demonstrated a correlation between CA7 expression and cell differentiation, its potential involvement in differentiation needs to be carefully determined, for the decrease in CA7 expression in less differentiated tissues could also be the result of other factors that lead to dedifferentiation, rather than downregulation of CA7 being a contributing factor in dedifferentiation. Nevertheless, regardless of the mechanism, our data indicates that CA7 might be useful in the histopathological grading of CRC.

Intriguingly, correlation analyses with clinicopathologic features from the two independent cohorts unanimously revealed a significant association between decreased CA7 expression and increased lymph node metastasis, advanced TNM stage and increased patient death, indicating that CA7 might be negatively involved in CRC progression. Recently, Monti SM and his groups have demonstrated that, apart from its canonical catalytic action of carbon dioxide hydration, CA7 has the ability to protect cells against oxidative damage [29,30]. Given that oxidative stress has been implicated in the pathogenesis of a wide spectrum of human cancers including CRC [31-34], the reported protective role of CA7 against oxidative stress might support a tumor-suppressing function for this enzyme. However, whether or not CA7 plays a functional role in the tumorigenesis and progression of CRC remains to be determined. Further studies using gain-of-function and loss-of-function strategies are warranted to address this issue.

The most interesting findings of this study are from the survival analysis results. Reduced expression of CA7 was associated with shortened survival for CRC patients not only in the training cohort but also in the external validation cohort. In univariate analysis, CA7 protein emerged as a significant prognostic factor of clinical outcome. Moreover, in multivariate analysis, it emerged as a significant independent predictor of survival in addition to tumor stage and patient age. The present study indicated that TNM stage also is an important prognostic factor in CRC, which is in agreement with its well established adverse prognostic effect [21]. Generally speaking, CRC patients who had early stage tumors (stages I-II) have a relatively favorable prognosis than those who had advanced stage tumors (stages III-IV). However, a subgroup of patients with early stage tumors have an increased risk of early recurrence and death. The potential mechanisms for these aggressive forms of early stage tumors are complicated; nevertheless, identifying this high-risk subgroup of patients would be of particular importance in the selection of patients for appropriate treatment. To further determine the prognostic value of CA7 in the therapeutic decision-making, we performed survival analyzes stage by stage. Importantly, reduced CA7 expression significantly predicted poor postoperative prognosis of patients with early stage tumors in both cohorts. More importantly, stage-based multivariate analyses from the two cohorts unanimously confirmed that decreased expression of CA7 also was an independent unfavorable prognostic indicator for patients with early stage tumors. Although we also observed a significant association between decreased CA7 expression and poor DSS in the advanced-stage patient group from the training cohort, its prognostic performance did not persist in the patients of the same stage category from the validation cohort (Figure 3D and Table 4). However, additional larger validations will be needed to further assess the prognostic significance of CA7 expression in patients with advance stage tumors. Of note, in contrast to our results, Bootorabi F *et al.* reported that high CA7 expression correlates with poor prognosis of patients with astrocytomas [16]. The discrepant results on the prognostic value of CA7 in different malignances indicate that its prognostic implication may be tissue-dependent and varies with the type of malignancy. The underlying mechanism for the prognostic importance of CA7 in CRC is currently unknown and needs to be further investigated. Nevertheless, our results suggest that determination of the intratumoral CA7 expression status may help to identify patients with aggressive forms of CRCs and further guide individualized therapy choices.

The present study remains to be improved on several aspects. Although we enrolled consecutive patients in the training cohort, some of them were lost to follow-up, which may introduce a bias. In addition, disease recurrence monitoring was incomplete in the two patient cohorts, resulting in the loss of disease-free survival data, which is also very important for a biomarker validation. Further prospective studies using large cohorts are necessary to validate the robustness of our findings before clinical translation.

## Conclusions

In summary, this study is the first to demonstrate that CA7 is frequently downregulated in CRC and that decreased expression of CA7 is closely related to aggressive clinical features and poor postoperative prognosis of CRC patients. Our findings from two independent cohorts provide evidence for the potential utility of CA7 as a prognostic marker for patients with CRC, especially for those with early stage tumors. In addition, results from the present work encourage further investigation of its potential role in CRC pathobiology.

## Additional files

Additional file 1:
**Distribution of the continuous variables of the two study cohorts.**

Additional file 2:
**Percentage of patients with different staining intensity of CA7 in the tumor or adjacent normal tissues in the validation cohort.**

## Abbreviations

CRC: Colorectal carcinoma
CA7: Carbonic anhydrase VII
DSS: Disease-specific survival
qPCR: Quantitative polymerase chain reaction
IHC: Immunohistochemistry
IRS: Immunoreactive score
