# Supplementary material for: Prognostic value of carbonic anhydrase VII expression in colorectal carcinoma
Source: BMC Cancer. 2015 Apr 1;15:209. doi: 10.1186/s12885-015-1216-y (PMC4406128; doi:10.1186/s12885-015-1216-y)
Supplement: Additional file 1: — Distribution of the continuous variables of the two study cohorts. [file 12885_2015_1216_MOESM1_ESM.doc]

**Additional file 1. Distribution of the continuous variables of the two study cohorts**

**Distribution of Continuous Variables of the Training Cohort (n=228).**

| **Variable** | **Median** | **Mean ± SEM** | **Range** | **Percentile** | |
| --- | --- | --- | --- | --- | --- |
|  |  |  |  | **25^th^** | **75^th^** |
| Age ( years) | 66.0 | 65.2 ± 0.7 | 24.0-90.0 | 58.0 | 74.0 |
| Tumor size (cm) | 5.0 | 5.5 ± 0.2 | 1.5-15.0 | 4.0 | 6.0 |
| DSS (months) | 75.5 | 60.4 ± 2.0 | 1.0-100.0 | 29.3 | 87.0 |

Abbreviations: SEM, Standard error of the mean; DSS, Disease-specific survival.

**Distribution of Continuous Variables of the Validation Cohort (n=151).**

| **Variable** | **Median** | **Mean ± SEM** | **Range** | **Percentile** | |
| --- | --- | --- | --- | --- | --- |
|  |  |  |  | **25^th^** | **75^th^** |
| Age ( years) | 65.0 | 65.1 ± 0.9 | 31.0-91.0 | 56.0 | 73.0 |
| Tumor size (cm) | 5.5 | 5.3 ± 0.2 | 1.0-14.0 | 3.5 | 6.0 |
| DSS (months) | 76.0 | 59.3 ± 2.5 | 1.0-96.0 | 28.0 | 86.0 |

Abbreviations: SEM, Standard error of the mean; DSS, Disease-specific survival.
