# Supplementary figures and images for: Prognostic value of carbonic anhydrase VII expression in colorectal carcinoma
Source: BMC Cancer. 2015 Apr 1;15:209. doi: 10.1186/s12885-015-1216-y (PMC4406128; doi:10.1186/s12885-015-1216-y)

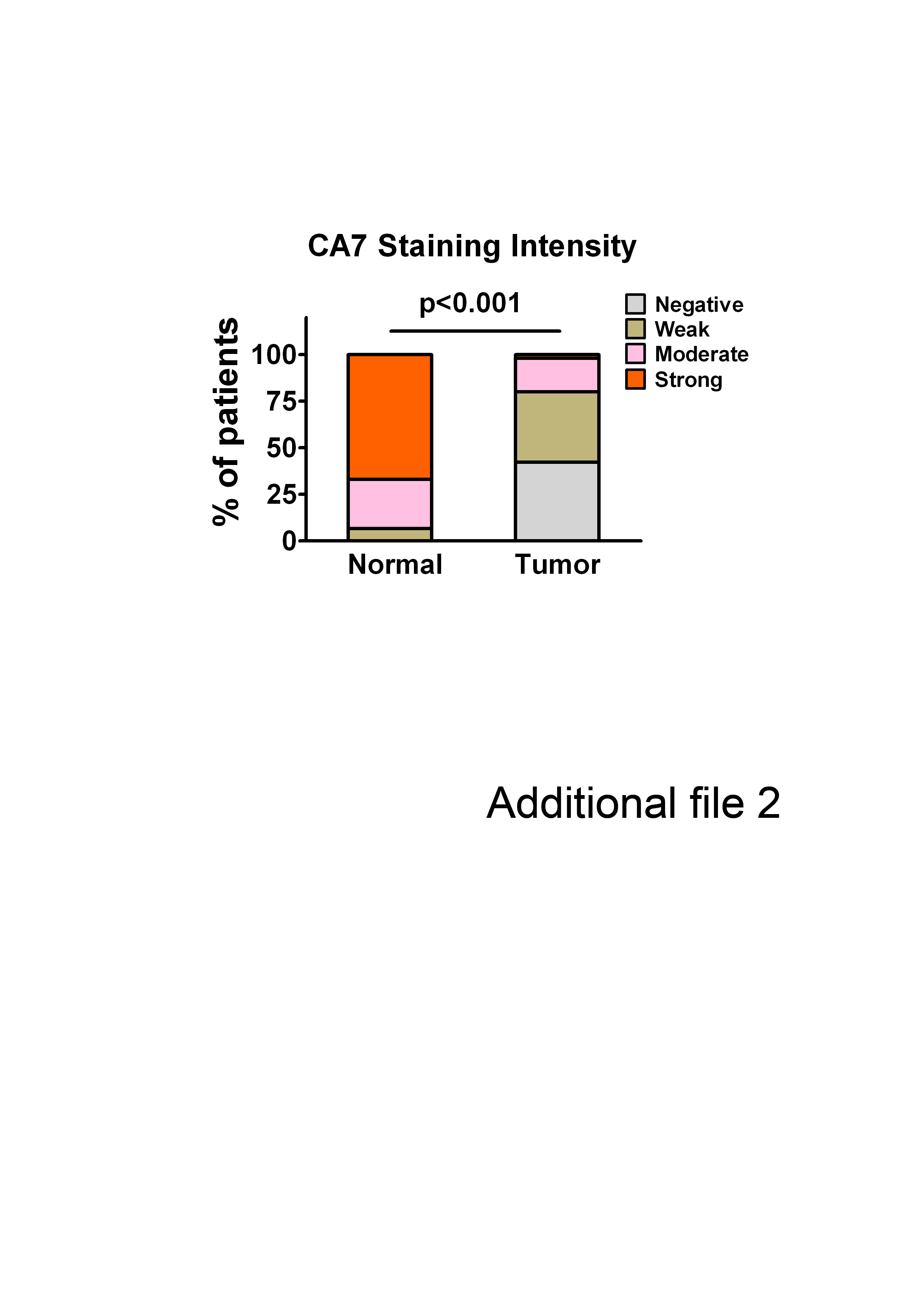

Supplement: Additional file 2: — Percentage of patients with different staining intensity of CA7 in the tumor or adjacent normal tissues in the validation cohort. [file 12885_2015_1216_MOESM2_ESM.jpeg]
